# Role of PI3K in myocardial ischaemic preconditioning: mapping pro‐survival cascades at the trigger phase and at reperfusion

**DOI:** 10.1111/jcmm.13394

**Published:** 2017-11-20

**Authors:** Xavier Rossello, Jaime A Riquelme, Sean M Davidson, Derek M Yellon

**Affiliations:** ^1^ The Hatter Cardiovascular Institute University College London London UK; ^2^ Advanced Center for Chronic Diseases (ACCDiS) Facultad de Ciencias Quimicas y Farmaceuticas & Facultad de Medicina Universidad de Chile Santiago Chile

**Keywords:** ischaemia/reperfusion injury, ischaemic preconditioning, cardioprotection, RISK pathway, SAFE pathway

## Abstract

The Reperfusion Injury Salvage Kinase (RISK) pathway is considered the main pro‐survival kinase cascade mediating the ischaemic preconditioning (IPC) cardioprotective effect. To assess the role of PI3K‐Akt, its negative regulator PTEN and other pro‐survival proteins such as ERK and STAT3 in the context of IPC, C57BL/6 mouse hearts were retrogradely perfused in a Langendorff system and subjected to 4 cycles of 5 min. ischaemia and 5 min. reperfusion prior to 35 min. of global ischaemia and 120 min. of reperfusion. Wortmannin, a PI3K inhibitor, was administered either at the stabilization period or during reperfusion. Infarct size was assessed using triphenyl tetrazolium staining, and phosphorylation levels of Akt, PTEN, ERK, GSK3β and STAT3 were evaluated using Western blot analyses. IPC reduced infarct size in hearts subjected to lethal ischaemia and reperfusion, but this effect was lost in the presence of Wortmannin, whether it was present only during preconditioning or only during early reperfusion. IPC increased the levels of Akt phosphorylation during both phases and this effect was fully abrogated by PI3K, whilst its downstream GSK3β was phosphorylated only during the trigger phase after IPC. Both PTEN and STAT3 were phosphorylated during both phases after IPC, but this was PI3K independent. IPC increases ERK phosphorylation during both phases, being only PI3K‐dependent during the IPC phase. In conclusion, PI3K‐Akt plays a major role in IPC‐induced cardioprotection. However, PTEN, ERK and STAT3 are also phosphorylated by IPC through a PI3K‐independent pathway, suggesting that cardioprotection is mediated through more than one cell signalling cascade.

## Introduction

Acute myocardial infarction (AMI) remains a leading cause of mortality worldwide 1. Timely reperfusion is the most effective treatment to limit myocardial infarct size (IS) and, consequently, to improve clinical outcome. Substantial effort has been made to implement early reperfusion therapy, which is crucial to salvage myocardial tissue, although paradoxically, injury still occurs as a consequence of reperfusion itself and remains an important target. Therefore, the development of novel therapeutic strategies to further reduce myocardial IS and preserve cardiac function 2 is still required.

IPC whereby brief cycles of coronary occlusion and reperfusion elicits protection was reported over 3 decades ago 3, 4 and has been replicated in all species examined 5, including humans 6, and has shown protection when applied to other organs and tissues 7. Importantly, other than reperfusion itself, IPC is considered the most powerful intervention available to protect the heart against myocardial ischaemia reperfusion injury (IRI) and has become the paradigm for cardioprotection 8.

IPC results in the recruitment of signalling pathways comprising protein kinases and phosphatases that converge on the mitochondria 7. The RISK pathway is considered the main pro‐survival kinase cascade mediating the IPC‐induced protective effect and encompasses two parallel signalling cascades: PI3K‐Akt and MEK1‐ERK1/2 8. Its activation not only mediates the protection induced by IPC, but also by other conditioning forms (pre‐, post‐, remote and pharmacological conditioning), and therefore appears to be a universal signalling paradigm for cardioprotection. Other alternative pathways, such as the Survivor Activator Factor Enhancement (SAFE) pathway comprising TNFα/STAT3, have also been proposed as IPC‐induced protection mediators 9.

A biphasic RISK activation response has been observed: occurring first during the preconditioning cycles (or ‘trigger’ phase) and then again during the first few minutes of reperfusion (‘mediator’ phase) 8, 10. Although the significance of the RISK pathways is generally accepted, the relative importance of the activation of these kinases and phosphatases, either at the trigger phase or exclusively at the mediator phase, remains to be fully elucidated. The activation of both RISK and SAFE pathways has been demonstrated to occur at these two time‐points in IPC studies 4, but there is a lack of comprehensive studies assessing the integrative or coordinated role of these signalling cascades. Cardioprotective signalling cascades have been mostly simplified in the literature to make its logical order more understandable. Crosstalk between the two components of the RISK pathway 11, 12 and between the RISK and the SAFE pathways 13, 14 have been described in only a few studies.

The aim of this study was to systematically characterize the role of PI3K‐Akt component of the RISK pathway, as well as its counter regulatory protein, PTEN in mediating the IPC cardioprotective effect during both the trigger phase and the reperfusion stage. In principle, both the activation (phosphorylation) of PI3K and the inactivation (also through phosphorylation) of PTEN should be expected to activate Akt and thus induce cardioprotection. We also aimed to further investigate the interplay between the PI3K‐Akt pathway and their parallel ERK and STAT3 cascades at both time‐points.

## Materials and methods

### Animals and chemicals

All procedures were performed in The Hatter Cardiovascular Institute, University College London, in strict accordance with the Home Office (United Kingdom) Guidance on Research and Testing using animals and the Animals (Scientific Procedures) Act of 1986.

Animals used were male C57BL/6 mice (9–12 weeks, 24–28 g weight), all of them obtained pathogen free from one supplier and housed under identical conditions.

Wortmannin, CAS 19545‐26‐7, the PI3 kinase inhibitor, was purchased from Merck Millipore (Nottingham, UK), and its concentration dose was chosen based on previous publications 13, 15. Dimethyl sulfoxide from BDH (Poole, UK) was used as the solvent for Wortmannin at a final concentration in the perfusion buffer of not more than 0.01%, as well as a vehicle control for the rest of the groups.

### Experimental design and study protocol

A total of 58 animals were used, although three hearts were excluded before randomization as they failed predefined exclusion criteria (see below). Therefore, 55 animals were randomly allocated to treatment groups in two separate experiments:

1. Study of infarct size. The effect on myocardial IS following IPC 4 cycles was studied using wortmannin 100 nM administered during the IPC protocol, or at reperfusion. The IPC protocol consisted of four cycles of 5 min. ischaemia and 5 min. reperfusion and was chosen based on previous publications demonstrating a reduction in myocardial IS 16, 17 (Fig. 1A).

**Figure 1:**
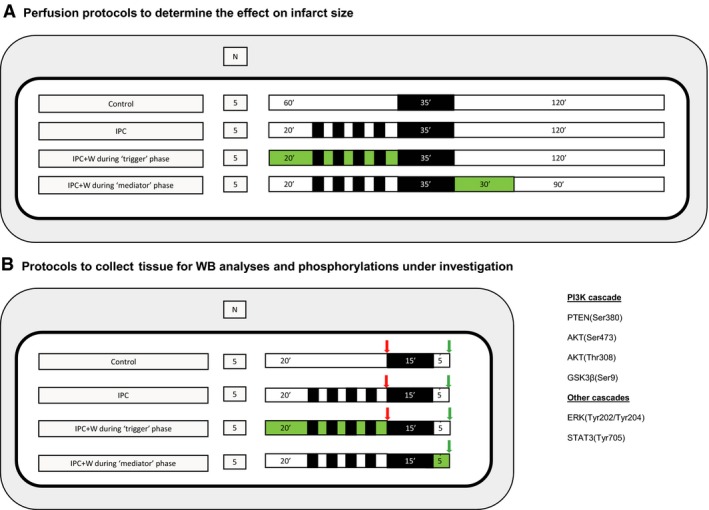
Study design and experimental protocols. Panel (**A**). Study design for IS determination protocols in the context of IPC and wortmannin (W) before and after index ischaemia. Four different experimental protocols were tested as follows: (*i*) control; (*ii*) IPC 4 cycles of 5 min. ischaemia and 5 min. reperfusion per cycle; (*iii*) IPC 4 cycles in the context of wortmannin administered during the stabilization period and IPC protocol; and (*iv*) IPC 4 cycles plus the administration of wortmannin upon reperfusion. Black boxes represent periods of ischaemia, white boxes represent periods of perfusion with Krebs–Henseleit buffer at 80 mm Hg, and green boxes represent the perfusion of wortmannin 100 nM. Panel (**B**). Study design to compare Akt and ERK phosphorylation analysis using Western blot. Four different experimental protocols were tested as follows: (*i*) control; (*ii*) IPC 4 cycles of 5 min. ischaemia and 5 min. reperfusion per cycle; (*iii*) IPC 4 cycles in the context of wortmannin administered during the stabilization period and IPC protocol; and (*iv*) IPC 4 cycles plus the administration of wortmannin upon reperfusion. Black boxes represent periods of ischaemia, white boxes represent periods of perfusion with Krebs–Henseleit buffer at 80 mm Hg, and green boxes represent the perfusion of wortmannin 100 nM. Arrows represent the moment where samples were collected (in red all samples collected after IPC protocol, known as trigger phase; in green all samples collected at reperfusion).
2. Study of phosphorylated protein levels. Activated levels of kinases and phosphatases involved in IPC‐induced cardioprotection were systematically studied in two sections, as summarized in Figure 1B. In the first, phosphorylated proteins were measured at the trigger phase in three treatment groups as follows: (*i*) control; (*ii*) IPC; and (*iii*) wortmannin plus IPC. In the second section, phosphorylated kinases were measured after 5 min. reperfusion in four treatment groups as follows: (*i*) control; (*ii*) IPC; (*iii*) IPC plus wortmannin during trigger phase; and (*iv*) IPC plus wortmannin during mediator phase.

In comparison with the protocols aimed at assessing myocardial IS, for those experiments aimed to evaluate protein phosphorylation levels, the period of ischaemia was reduced to 15 min. and reperfusion shortened to 5 min. Shorter protocols of IRI are well accepted in literature 17, 18 on the basis of (*i*) obtaining enough non‐necrotic tissue to evaluate kinase activation and (*ii*) evaluate the key moment where the protection is elicited by kinase activation and mPTP opening delay 19, 20.

The sample size for the evaluation of myocardial IS was estimated based on previous publications 16, whilst for Western blot analysis, the sample size was chosen in line with convention 21. Reproducible randomization sequences were used to allocate mice using stata version 13.1. (StataCorp, College Station, TX, USA).

### Langendorff isolated perfused mouse heart

Terminal anaesthesia and anticoagulation with an intraperitoneal injection of 60 mg/kg sodium pentobarbitone and 100 IU heparin were administered to mice, respectively. Hearts were then excised and immediately submerged in ice‐cold modified Krebs–Henseleit buffer (composed of 118 mmol/l NaCl, 25 mmol/l NaHCO3, 11 mmol/l glucose, 4.7 mmol/l KCl, 1.22 mmol/l MgSO4.7H20, 1.21 mmol/l KH2PO4 and 1.84 mmol/l CaCl2.2H20). Hearts were then cannulated with a 21‐gauge cannula and retrogradely perfused, on a murine Langendorff system, at 80 mm Hg pressure. Both heart isolation and Langendorff perfusion were carried out with filtered modified Krebs–Henseleit buffer aerated with a mixture of O2 (95%) and CO2 (5%) to maintain pH at 7.40 ± 0.3, as described previously 16.

Predefined exclusion criteria were as follows: (*i*) more than 4 min. time between heart excision and the beginning of perfusion in the Langendorff mode; (*ii*) temperature above or below the 37 ± 0.5°C range; and (*iii*) buffer flow rate of the isolated heart of <1 ml/min. or more than 6.5 ml/min. on the Langendorff preparation during the stabilization period. After assessing for exclusion criteria in an initial 20 min. stabilization period, hearts were subjected to the experimental protocols.

### Myocardial infarct size

Myocardial IS was assessed after global ischaemia and reperfusion by injecting 5 ml of 2,3,5‐triphenyl tetrazolium chloride in phosphate‐buffered saline through the aorta and incubating the heart for 10 min. at 37°C to delineate the infarcted (white) *versus* viable (red) tissue 22. Hearts were frozen overnight at – 20°C and sectioned perpendicularly to the long axis the day after. The heart slices were subsequently transferred into 10% formalin buffer for 1 hr. Images were taken and coded to blind the analyser. Planimetry analysis using Image J version 1.47 (NIH, Bethesda, Maryland, USA) was performed to accurately quantify the percentage of myocardial IS.

### Western blot analyses

Hearts prepared for Western blot analysis were collected following the protocols illustrated in Figure 1B and thereafter snap‐frozen in liquid nitrogen. The tissue was then stored at −80°C until further processing. The tissue was homogenized in protein lysis buffer, containing Tris pH 6.8 [100 nM], NaCl [300 Mm], NP40 0.5%, Halt protease inhibitor cocktail, Halt phosphatase inhibitor cocktail, 0.5 M EDTA (all from Thermo Scientific, Loughborough, UK) and adjusted to pH 7.4. Homogenates were then sonicated before being centrifuged for 10 min. at 10,000 g at 4°C to remove debris and DNA. Protein content were then determined using bicinchoninic acid (BCA) protein assay reagent (Sigma‐Aldrich, Gillingham, UK) and protein levels corrected accordingly to ensure equal protein loading. NuPAGE LDS Sample Buffer (4X) (Thermo Fisher Scientific) plus 5%β‐mercaptothanol were added, and the samples were denatured by heating to 100°C for 10 min. Samples were loaded on NuPAGE Novex 10% Bis‐Tris protein gels (Thermo Fisher Scientific, Loughborough, UK) using the Mini Protean III system (Bio‐Rad, Watford, UK). Proteins were electro‐transferred onto nitrocellulose blotting membrane (GE Healthcare Life Science, Amershamm UK) using wet transfer in a Bio‐Rad Mini Trans‐Blot. The membranes were blocked by incubating in 5% bovine serum albumin/PBS tween and subsequently incubated with appropriate primary antibodies at 4°C overnight.

Primary antibodies used were acquired from Cell Signaling Technology (Leiden, The Netherlands): Akt (#9272), Phospho‐Akt (Ser473) (#9271), Phospho‐Akt (Thr308) (#2965), ERK1/2 (#9102), Phospho‐ERK1/2 (Thr202/Tyr204) (#9101), GSK‐3β (#9315), Phospho‐GSK‐3β (Ser9) (#5558), Stat3 (#9139), Phospho‐Stat3 (Tyr705) (#9145), Phospho‐PTEN (Ser380) (#9551) and PTEN (#9552). Anti‐GAPDH (mAbcam, #9484) was used as loading control. The day after, membranes were probed with secondary infrared fluorescence antibodies. Levels of protein were quantified using the Odyssey imaging system from Li‐Cor Biosciences (Image Studio Lite Ver 5.2, Cambridge, UK).

### Statistical analyses

Normal distribution of each data subset was tested using graphical methods and the Kolmogorov–Smirnov method. All values are presented as mean ± standard error of the mean. If normally distributed, continuous data were compared using one‐way analysis of variance followed by *post hoc* pairwise comparisons to the control group using the Dunnett's test. If highly skewed distributed, the nonparametric Kruskal–Wallis test was used with subsequent post hoc pairwise comparisons to the control group adjusted by the Dunn's test. A *P* value of <0.05 was considered statistically significant. stata software, version 13.1 (StataCorp) and GraphPad Prism version 6.00 (GraphPad Software, La Jolla, CA, USA) were used to perform both the analysis and the graphics. The results were reported according to the ARRIVE guidelines for reporting animal research 7.

## Results

### PI3K mediates the IPC protective effect against myocardial IRI

**Figure 2:**
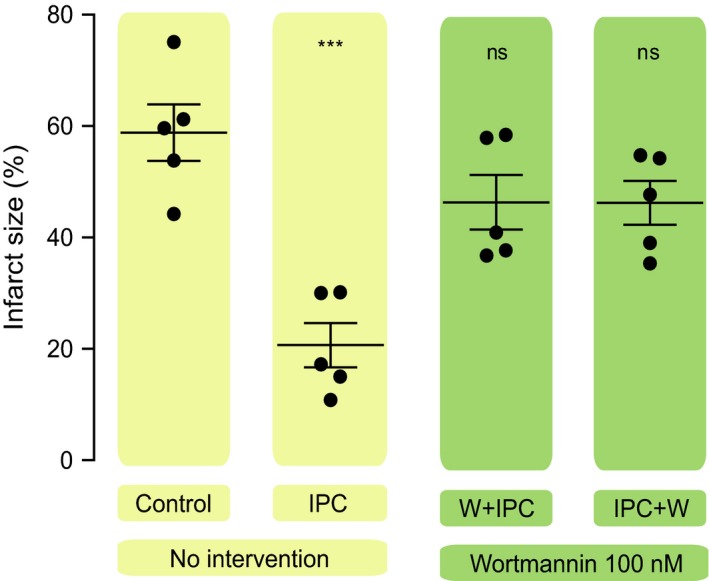
Role of PI3K in the protective effect of IPC at the trigger phase and at reperfusion. Scatter dot blots: black lines represent mean ± S.E.M., and circles represent individual animal data. Myocardial infarct size was significantly smaller with IPC compared to control group and either the administration of Wortmannin before or after ischaemia index abolished the cardioprotective effect of IPC.

To investigate the role of the PI3K‐AKT cascade in IPC‐induced cardioprotection, isolated perfused mouse hearts were subjected to 35 min. global ischaemia, followed by 2‐hr reperfusion. IPC resulted in reduction in IS compared with the control group (21 ± 4 *versus* 59 ± 5%, *P* < 0.001) (Fig. 2). Administration of wortmannin either during the trigger phase of the IPC protocol or during the mediator phase abrogated its IS‐limiting effect when compared to control group (46 ± 5%, *P* = 0.158; and 46 ± 4, *P* = 0.154, respectively) (Fig. 2).

### PI3K mediates IPC‐induced activation of Akt

**Figure 3:**
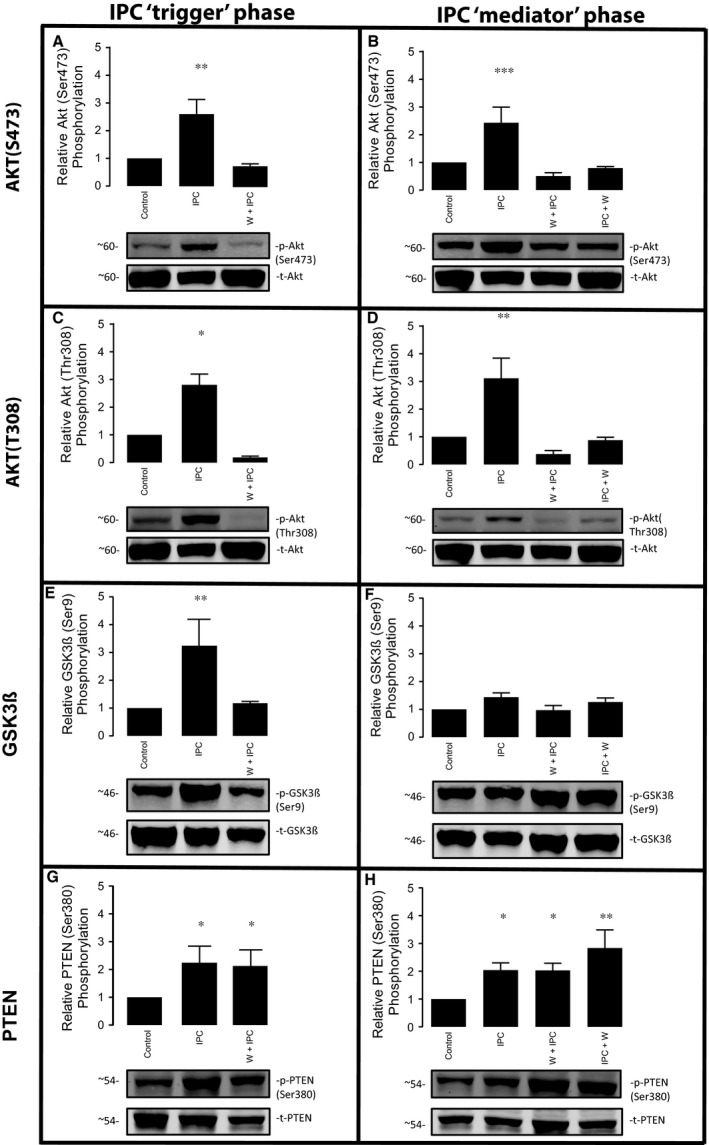
Impact of PI3K inhibition in IPC activated signalling cascades (Akt phosphorylations are depicted in panels A to D; GSK3β in panels E and F; and PTEN in panels G and H). Bar graph shows the percentage of phosphorylation in all groups compared to the control group, expressed as mean ± S.E.M. (percentage of relative phosphorylation), *n* = 5 per group.

To confirm the activation of Akt during the IPC trigger phase and the early phase of reperfusion both of the main Akt phosphorylation sites (Ser‐473 and Thr‐308) were studied. As expected, IPC increased Akt phosphorylation in the trigger phase at both sites (Ser‐473: 2.6 ± 0.5‐fold, *P* = 0.010 *versus* control; and Thr‐308: 2.6 ± 0.5‐fold *versus* control, *P* = 0.034), and phosphorylation was prevented when wortmannin was applied during the IPC trigger phase (Fig. 3A and C). Likewise, IPC increased the phosphorylation of Akt during the mediator phase of reperfusion (Ser‐473: 2.4 ± 0.6‐fold *versus* control, *P* < 0.001; and Thr‐308: 3.1 ± 0.7‐fold *versus* control, *P* = 0.001). However, this phosphorylation was consistently abrogated when wortmannin was administered either during the trigger or mediator phase (Fig. 3B and D).

### IPC phosphorylates GSK3β at the trigger phase, but not the mediator phase

To explore the downstream targets activated by PI3K‐Akt both at the trigger phase and upon reperfusion, we investigated the role of the serine/threonine kinase GSK3β. In contrast to many protein kinases, GSK3β is active in resting cells and inactivated by phosphorylation 23. On stimulation, GSK3β is phosphorylated at serine 9, resulting in inhibition of its kinase activity. As illustrated in Figure 3E, IPC caused an increase in GSK3β phosphorylation during the trigger phase (3.2 ± 0.9‐fold *versus* control, *P* = 0.007), which was blocked by wortmannin. On the contrary, GSK3β was not significantly phosphorylated at reperfusion in preconditioned hearts compared to control group (1.4 ± 0.2‐fold *versus* control, *P* = 0.742). Moreover, the administration of wortmannin either during the trigger phase or mediator phase did not affect GSK3β phosphorylation levels when measured at reperfusion (Fig. 3F).

### IPC phosphorylates PTEN through a PI3K‐independent mechanism

PTEN is a phosphatase that counter regulates the PI3K/AKT signalling pathway 24. While phosphorylation by PI3K stimulates Akt activity, PTEN dephosphorylates and down‐regulates Akt activity. The phosphorylated form of PTEN is considered inactivated and is therefore a major negative regulator of the RISK pathway 25. PTEN phosphorylation was significantly increased immediately following an IPC stimulus (2.2 ± 0.6‐fold *versus* control, *P* = 0.022). Interestingly, this was not affected by the administration of wortmannin (Fig. 3F). IPC was also associated with an increase in phosphorylated PTEN during the mediator phase (2.0 ± 0.3‐fold *versus* control, *P* = 0.042), and again, this was not affected by the presence of wortmannin during either the trigger or mediator phases (Fig. 3H).

### IPC‐induced ERK activation involves PI3K during the trigger phase, but not at reperfusion

**Figure 4:**
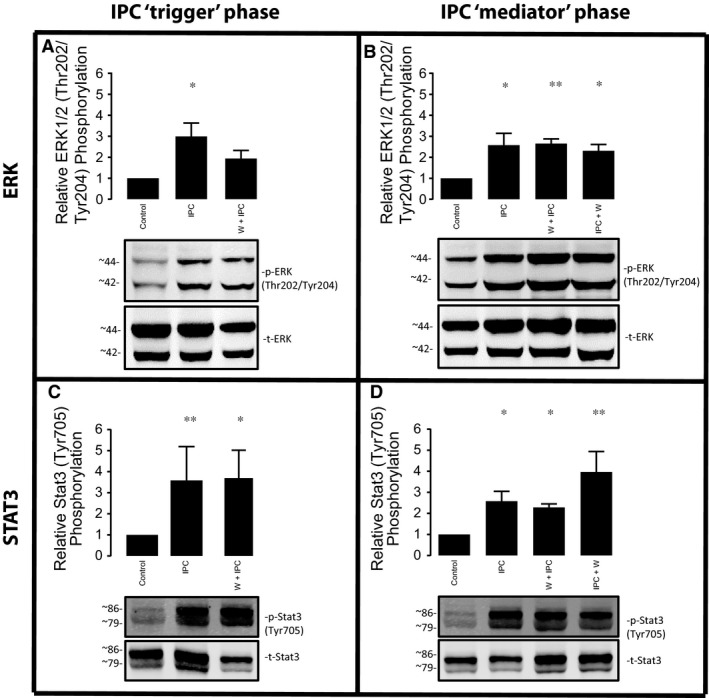
Impact of PI3K inhibition in IPC activated ERK (panels A and B) and STAT3 (panels C and D). Bar graph shows the percentage of phosphorylation in all groups compared to the control group, expressed as mean ± S.E.M. (percentage of relative phosphorylation, normalized by its total), *n* = 5 per group.

During the IPC trigger phase, phosphorylated levels of ERK were significantly increased by IPC (3.0 ± 0.6‐fold times compared to control, *P* = 0.016). This was partially abrogated by wortmannin, such that there was no longer a significant increase in ERK phosphorylation during the trigger phase after IPC (Fig. 4A). IPC also caused a significant increase in ERK phosphorylation during the mediator phase (2.6 ± 0.6‐fold increase for IPC, *P* = 0.016), but in contrast to the response observed in the trigger phase, this was unaffected by the administration of wortmannin (IPC + wortmannin during trigger phase: 2.7 ± 0.2‐fold, *P* = 0.009; IPC + wortmannin during the mediator phase: 2.3 ± 0.3‐fold, *P* = 0.031) (Fig. 4B).

### IPC activates STAT3 through a PI3K‐independent mechanism

The SAFE pathway, which involves TNFα, JAK and STAT3, has been described as an alternative RISK‐independent cascade that may be important for mediating the cardioprotective effect elicited by IPC in some circumstances 13, 26. We found that IPC significantly increased levels of phosphorylated STAT‐3 (Tyr705) during the trigger phase (3.6 ± 1.6‐fold *versus* control, *P* = 0.009) (Fig. 4C). The administration of wortmannin during the trigger phase did not alter levels of phosphorylated STAT3. Similarly, IPC increases levels of phosphorylated STAT3 in the mediator phase (2.6 ± 0.5‐fold *versus* control, *P* = 0.049), and this was unchanged by the administration of wortmannin during either the trigger or mediator phase (Fig. 4D).

## Discussion

**Figure 5:**
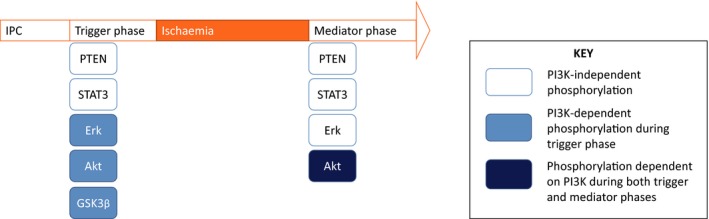
Summary of our findings.

In our initial experiments, we confirmed previous observations that PI3K activity is required during both the trigger phase and mediator phase in order for IPC to reduce the infarct size. As expected, IPC increased the levels of Akt phosphorylation during both the trigger and mediator phases, as summarized in Figure 5. Interestingly, Akt phosphorylation during both phases was completely abrogated by PI3K inhibition during just the trigger phase or the mediator phase, suggesting the existence of a memory effect. In contrast, one of the kinases downstream of the PI3K/Akt pathway, GSK3β, was phosphorylated only during the trigger phase after IPC. PTEN was phosphorylated during both the trigger and mediator phases after IPC, but this was independent of PI3K. IPC increases ERK phosphorylation during both phases, but was only PI3K‐dependent during the trigger phase. Finally, STAT3, the kinase mediator of the SAFE pathway, was activated by IPC in both the trigger phase and mediator phase, and this phosphorylation was independent of PI3K activity.

### Role of PI3K in the protective effect of IPC at the trigger phase and at reperfusion

IPC protects against myocardial IRI by activation of the RISK pathway 10, 15. Our data confirm the pivotal role of PI3K as mediator of IPC, as its pharmacological inhibition either during the trigger phase or at reperfusion abolished the IS‐limiting effect provided by IPC. Wortmannin is a cell‐permeable fungal metabolite that acts as a selective and irreversible inhibitor of the PI3K catalytic activity that has been widely used in pharmacological cardioprotection studies, but similar results have also been demonstrated using the reversible inhibitor LY294002 15.

The fundamental concept of the RISK pathway may be summarized in two ways: (*i*) short‐term activation of these kinases is protective triggering pro‐survival pathways, whilst long‐term activation may have detrimental effects such as cell growth e.g hypertrophy; and (*ii*) their activation occurs both during the preconditioning phase 27 and at reperfusion 20. It has been well established that these two phases are crucial to mediate protection, as both of them can be pharmacologically intervened 28. Taking into account that IPC signalling architecture can be extrapolated to most cardioprotective therapies 29, we focused on thoroughly dissecting the key signalling events occurring at both phases when abolishing PI3K phosphorylation.

PI3K‐Akt, one of the parallel cascades involving the RISK pathway, has been described to activate a series of events. The activation of PI3K by IPC promotes the phosphorylation of PDK1, which in turn activates Akt to subsequently recruit a wide range of pro‐survival downstream targets such a GSK3β, p70s6k and eNOS 25. On the contrary, PTEN counter regulates the action of PI3K by dephosphorylating its product phosphatidylinositol(3,4,5)phosphate (abbreviated as PIP3). The activation of the PI3K/Akt pathway inhibits mitochondrial permeability transition pore (MPTP) opening, which is considered the major downstream end‐effector of the RISK pathway 10, 30.

### Impact of PI3K inhibition in IPC activated signalling cascades

Akt is a serine/threonine protein kinase. Its activity is primary controlled by PI3K and PTEN through the modulation of PIP3 levels. Full activation of Akt happens through the phosphorylation of a Thr‐308 residue in the catalytic domain by PDK1 and a Ser‐473 residue by mTOR2, whilst its inactivation is mediated through dephosphorylation of the two regulatory sites by the serine‐threonine phosphatase PP2A. Akt is widely used as surrogate marker for PI3K activation 31 based on the assumption that its readout is proportional to PIP3 levels. However, both PIP3 levels and Akt activity can be the result of both PI3K activation and PTEN inactivation and this fact needs to be taken into account when interpreting our results. In our study, Akt phosphorylation at both residues was abrogated in all preconditioned hearts treated with wortmannin. Therefore, in preconditioned hearts treated with the PI3K inhibitor, the abolishment of IS‐sparing effect was mirrored by lack of Akt phosphorylation.

GSK3β is a serine/threonine kinase that has been proposed to be the downstream point of convergence of the RISK pathway, which when phosphorylated (and thus inhibited) at serine‐9 by Akt in response to PIP3 increase 32, thus promoting inhibition of MPTP opening 33, enhancing cell survival. 23. Our data suggest that GSK3β is phosphorylated at the trigger phase as a consequence of PI3K activation, but this status is lost at the early reperfusion stage. In agreement with our results, *Tong et al*. 32 have reported increased phosphorylated levels of GSK3β following an IPC protocol, which were blocked by wortmannin. Moreover, pre‐treatment with GSK3β inhibitors also mimicked the protective effect of IPC through a reduction on myocardial IS 32. In another study, pharmacologic and genetic ablation of GSK3β failed to abrogate the protective effect conferred by IPC 33. Similarly, mice with a knockin of GSK3 beta mutated at ser9 and ser21 remained amenable to protection by IPC 23. In that study, both insulin and cyclosporin A were effective in delaying mitochondrial permeability transition pore opening in adult cardiac myocytes from GSK3β knockin mice, therefore, suggesting that mice lacking functional GSK3β can be protected through the insulin‐PI3K‐Akt‐mPTP axis, without the intervention of GSK3β. Overall, it appears that inhibition of GSK3β may play an important role during the IPC protocol, but not afterwards. However, future studies need to clarify the contentious role of GSK3β in cardioprotection.

The activation of pro‐survival protein kinases has been studied in great detail in the context of preconditioning. However, little is known about the role of the phosphatases in this setting. Inhibition of phosphatases (PP1 and PP2A) during the preconditioning phase have been shown to abolish the protective effect of preconditioning, whilst its activation during reperfusion improved protection in preconditioned hearts 34. The ability to up‐regulate the RISK pathway by the use of phosphatase inhibitors during the early reperfusion phase is considered an attractive and unexplored approach to limiting IRI.

PTEN is a dual lipid and protein phosphatase that antagonizes PI3K/AKT signalling pathway. Whereas PI3K activity results in an increased production of the second messenger PIP3 to activate the pro‐survival downstream cascade, PTEN dephosphorylates PIP3 to PIP2 to down‐regulate Akt activation. The phosphorylation form of PTEN is considered inactive. After dephosphorylation, PTEN is activated but is also degraded rapidly as its half‐life is substantially reduced 35. Therefore, the phosphorylation (inactivation) of PTEN should be expected to induce cardioprotection following IRI, whilst its dephosphorylated (activated) status should be expected to be detrimental 36, 37. Interestingly, our results suggest that IPC induces inhibition of negative regulator PTEN. This observation is in line with the study of Cai and Semenza who identified a reduction in the activity of PTEN following IRI in an isolated perfused rat heart model 35. Furthermore, PTEN haploinsufficiency mice have been shown to reduce the threshold of protection in IPC 38. This result suggests that IPC not only activates pro‐survival kinases but also inhibits their major counter regulators (i.e. PTEN, PP1 and PP2A). Additionally, IPC‐mediated PTEN phosphorylation appears to be independent of PI3K, suggesting that both activation of PI3K and inactivation of PTEN can work in unison, which may suggest that pharmacological intervention of both proteins at the same time may produce synergistic effect in the context of cardioprotection. Further studies need to be undertaken to explore this hypothesis. It is time for PTEN to gain further attention and respect in cardioprotection, as it still remains an unknown player despite its crucial role in regulating Akt.

### Impact of PI3K inhibition in IPC activated ERK and STAT3

Cardioprotective signalling has been described as highly interactive 29. Within the RISK pathway, some studies have suggested crosstalk between the PI3K‐Akt and the ERK1/2 cascades, demonstrating that inhibiting one cascade activates the other one and vice versa 12. Our study shows that ERK is phosphorylated following an IPC stimulus, but partially inhibited after the administration of the PI3K inhibitor wortmannin. These results may suggest a PI3K predominance during the IPC early trigger phase, although they contrast with the lack of effect of the PI3K inhibitor on ERK phosphorylated levels at reperfusion. In this context, a previous study from our laboratory using a pharmacological approach with specific PI3K and ERK inhibitors in the isolated perfused rat heart model suggested that the crosstalk between PI3K/Akt and ERK is not balanced, with the PI3K cascade playing a more determinant role in mediating cellular survival 12.

The SAFE pathway, which involves TNFα, JAK and STAT3, has been described as an alternative RISK‐independent cascade mediating the cardioprotective effect elicited by IPC. The cardioprotective effect of STAT3 is believed to be partly related to its translocation to the mitochondria by modulating respiration and inhibiting the mPTP opening 39. Like its RISK counterpart, the SAFE pathway is also activated during both the IPC stimulus and the early phase of reperfusion to protect the heart against IRI 9, 13. We found that STAT3 is activated following an IPC protocol, and this is unaffected by the inhibition of PI3K. Interestingly, upon administration of wortmannin, the protective effect elicited by IPC was lost despite the STAT3 pathway being activated. Our results differ from those described by Lecour *et al*. 13 in adult mouse cardiomyocytes, in which wortmannin administered during the IPC trigger phase decreased STAT‐3 phosphorylation and abolishing the protection afforded by IPC. However, in our study, the mean infarct size for hearts treated with PI3K inhibitor is approximately in a half‐way point between the preconditioned and the non‐preconditioned hearts. This difference has been enough to abolish statistical significance but leaves further room to speculate on the involvement of more than one signalling cascade to promote maximal protection, as it has been demonstrated that using a STAT‐3 inhibitor also abolishes the protective effect of IPC 13. Rather than contradict, our results seem to complement the concept of having multiple protective pathways, namely both RISK and RISK‐independent pathways. This may be important in establishing a cardioprotective stratagem that is effective in patients 2.

In the light of our data, we hypothesize that PI3K has a dominant role to mediate the IPC protective effect. On close examination, the activation of the p85 regulatory subunit of PI3K has been demonstrated to control the serine phosphorylation of STAT3, a critical step for the formation of stable STAT3 homodimers 40. In other contexts, STAT3 activation has also been shown to be dependent on PI3K recruitment 41, thus suggesting that PI3K can regulate STAT3 phosphorylation. Despite this evidence supporting a predominant role for PI3K in IPC, other alternative pathways have been demonstrated to be involved in a RISK‐independent manner. Hence, cardiac‐specific STAT3 deficient mice have been unable to show Akt phosphorylation following an IPC stimulus, and the pharmacologic preconditioning induced by TNFα have demonstrated protection through STAT3 phosphorylation without involving PI3K activation 9. To our understanding, the activation of both the RISK and the SAFE pathway can occur concomitantly either in IPC or in pharmacologic conditioning 14. It is also unknown whether activation of the two pathways provides an additive effect to maximize the protection.

### Limitations of the study

Only one PI3K inhibitor was used, and its effect when administered alone was not tested, although many previous studies have shown that wortmannin does not have major effects on IS and protein phosphorylation in the absence of IPC 15, 18. Wortmannin may inhibit other kinases such as myosin light chain kinase or PI 4‐kinase at concentrations higher than that required for inhibition of PI3K. With regard to protein analyses, not all phosphorylated residues were studied here—that is PTEN possesses three phosphorylation sites (Ser380, Thr382 and Thr383) and STAT3 can be phosphorylated at both its serine 727 and tyrosine 705 residues. Of note, we lack results involving other important mediators in the IPC‐induced molecular signalling, such as PKC—many other kinases and proteases remain to be explored in this setting.

Our results are based on the assumption that Akt phosphorylation is the result of the activation of PI3K by IPC, but we have no direct evidence that IPC increases PI3K activity in the heart. However, we have also shown an IPC‐mediated PTEN phosphorylation that appears to be independent of PI3K and could also result in the increase in Akt phosphorylation levels. In this study, we cannot distinguish between the amount of Akt phosphorylated by PI3K activation or PTEN inactivation, although we have shown that wortmannin abolishes both the protective effect of IPC and the phosphorylation of Akt. Therefore, caution is needed when interpreting the source of Akt activation affords by IPC, as PIP3 levels are regulated by both PI3K and PTEN. Further studies need to be undertaken to explore the actual contribution of the kinase PI3K and the phosphatase PTEN to the rise of PIP3 and subsequent Akt activation.

An additional limitation of the study is that we did not use functional assessment of the heart to try and avoid potential manipulation injury, as it has been shown that an intraventricular balloon can influence the salvage pathways being investigated 42.

### Summary and conclusions

In summary, PI3K activity is required during both the trigger and mediator phases for IPC to limit the infarct size. IPC increased the levels of Akt phosphorylation during both the trigger and mediator phases and this effect was fully abrogated by PI3K inhibition in both phases, whilst its downstream GSK3β was phosphorylated only during the trigger phase after IPC. Both PTEN and STAT3 were phosphorylated during both the trigger and mediator phases after IPC, but this was independent of PI3K. IPC increase ERK phosphorylation during both phases, being only PI3K‐dependent during the trigger phase.

In conclusion, the RISK pathway is recruited not only by IPC, but also by other agents and its activation at early reperfusion is considered a unifying pattern of cardioprotective signalling 8. Further elucidation of the signalling pathways behind the IPC‐induced protective effect is expected to reveal targets for cardioprotection, which can be manipulated by pharmacological agents to benefit patients undergoing acute myocardial infarction 2.

## Author contributions

X.R. contributed to conception and design, acquisition, analysis and interpretation of the vast majority of the experiments, drafted and critically revised the manuscript and agreed to be accountable for all aspects of work ensuring integrity and accuracy. Dr J.A.R. contributed to the interpretation of the experiment and critically revised the content of the manuscript. Dr S.D. and Professor D.M.Y. critically revised the manuscript and approved its final version.

## Conflict Of interest

The authors declared no conflict of interest.
